# Associations of triglyceride–glucose–body mass Index and C-reactive protein with isolated nocturnal hypertension: evidence from a retrospective observational study

**DOI:** 10.3389/fcvm.2026.1770278

**Published:** 2026-03-05

**Authors:** Cagdas Kaynak

**Affiliations:** Depatment of Cardiology, Siirt Education and Research Hospital, Siirt, Türkiye

**Keywords:** ambulatory blood pressure monitoring, c-reactive protein, insulin resistance, isolated nocturnal hypertension, triglyceride–glucose–body mass index

## Abstract

**Objective:**

Isolated nocturnal hypertension (INH) is an underrecognized blood pressure (BP) phenotype that is associated with a greater burden of cardiovascular risk despite normal daytime BP values. Metabolic disturbances together with sustained low-level inflammatory activity have been implicated in circadian BP abnormalities; however, data regarding simple and widely available biomarkers for identifying INH remain limited. The aim of the present study was to explore the relationships between metabolic indices and inflammatory markers in the context of INH and to further evaluate their potential value in predicting this condition.

**Methods:**

We conducted a retrospective observational analysis at a single institution, including individuals who were evaluated using 24-hour ambulatory blood pressure monitoring (ABPM) between March 2024 and March 2025. INH was defined by a mean daytime BP below 135/85 mmHg in the presence of a mean nighttime BP of at least 120/70 mmHg. Demographic characteristics, laboratory parameters, C-reactive protein (CRP) levels and several metabolic indices—namely the triglyceride–glucose (TyG) index, its body mass index–adjusted form (TyG-BMI), and the atherogenic index of plasma (AIP)—were included in the analysis. Independent determinants of INH were examined using logistic regression models, while diagnostic accuracy was evaluated through receiver operating characteristic (ROC) curve analysis.

**Results:**

The study population consisted of 241 participants, including 137 individuals with INH and 104 normotensive controls. Compared with the control group, those with INH exhibited significantly elevated TyG index, TyG-BMI, AIP and CRP levels (all *p* < 0.001). In multivariate logistic regression analysis, TyG-BMI [odds ratio [OR] 1.030, 95% confidence interval [CI] 1.002–1.059; *p* = 0.034] and CRP (OR 3.878, 95% CI 2.609–5.763; *p* < 0.001) remained independently associated with INH. ROC analysis demonstrated moderate discriminatory ability for TyG-BMI [area under the curve (AUC) 0.696, *p* < 0.001] and high discriminatory performance for CRP (AUC 0.861, *p* < 0.001).

**Conclusion:**

Metabolic dysregulation and systemic inflammation are strongly associated with INH. TyG-BMI and CRP, derived from routinely available clinical data, may act as practical markers to identify individuals at increased risk for this clinically important BP phenotype.

## 1 Introduction

Globally, hypertension (HT) remains a major preventable factor influencing cardiovascular disease development and related mortality (1). Beyond office measurements, ambulatory blood pressure monitoring (ABPM) confers a thorough evaluation of circadian blood pressure (BP) patterns and identifies clinically significant phenotypes that are often missed in conventional therapy (2). Among these, isolated nocturnal hypertension (INH) represents a distinct and increasingly recognized entity.

The INH refers to an increase in nighttime BP (≥ 120/70 mmHg) while maintaining normal daytime BP (< 135/85 mmHg) and the diagnosis of this condition necessitates the use of ABPM (1, 2). Nighttime BP has repeatedly demonstrated greater predictive value for cardiovascular events and all-cause mortality than daytime or office BP values (3), underscoring the prognostic significance of this phenotype.

Subjects diagnosed with INH exhibit a greater occurrence of left ventricular hypertrophy, renal dysfunction and subclinical cerebrovascular damage, even in the absence of overt daytime HT, and this phenotype has also been associated with increased carotid intima–media thickness, reflecting early vascular remodeling and potentially elevating long-term cardiovascular risk (4). The occult nature of the condition often leads to delayed diagnosis and underestimation of cardiovascular risk, further emphasizing the indispensable role of ABPM for early risk stratification (2).

The underlying mechanisms of INH are multifaceted and remain incompletely understood. Suggested mechanisms encompass disturbance of autonomic balance, dysfunction of the endothelium, improper sodium regulation and disruption of central and peripheral circadian regulation (4, 5). Importantly, these mechanisms converge on metabolic and inflammatory pathways that influence nocturnal BP regulation (5).

Insulin resistance (IR) is a key contributor to vascular impairment and the pathogenesis of hypertension (6). Due to the impracticality of direct measurements of IR for regular application, surrogate indices obtained from standard laboratory parameters have garnered interest. Importantly, the triglyceride–glucose (TyG) index has gained recognition as a simple and validated substitute marker of IR and has been linked to arterial stiffening, endothelial impairment and the development of atherosclerotic disease (7, 8). In population-based studies, elevated TyG levels have been associated with incident HT and increased cardiovascular risk (6, 9).

Building on TyG, the triglyceride–glucose–body mass index (TyG-BMI) composite index has been proposed to integrate metabolic dysregulation with adiposity, a key determinant of IR and cardiometabolic burden (10). Accumulating evidence suggests that this composite index may outperform TyG alone in predicting metabolic syndrome, type 2 diabetes and cardiovascular morbidity (11).

Alongside metabolic dysregulation, chronic low-grade inflammation is a fundamental pathway in HT and atherosclerosis. C-reactive protein (CRP) has been widely reported to correlate with endothelial dysfunction, arterial stiffness, and adverse cardiovascular outcomes (12, 13). Inflammatory activation contributes to nocturnal BP elevation through vascular impairment, heightened sympathetic activity, and altered renal sodium handling (4, 13). However, the specific contribution of readily measurable inflammatory markers to INH has not been adequately characterized.

Despite increasing recognition of INH as a high-risk BP phenotype, data regarding simple, widely available metabolic and inflammatory biomarkers remain limited. Most existing studies have focused on traditional risk factors, whereas composite indices reflecting metabolic stress and inflammation have received comparatively little attention in relation to nighttime BP behavior.

Therefore, this study aimed to evaluate whether routinely obtained metabolic indices and inflammatory markers are associated with INH and to examine their potential value in facilitating the detection of individuals at heightened risk for this clinically relevant BP phenotype.

## 2 Materials and methods

### 2.1 The study design and population

Healthcare records from the Cardiology Department of Siirt Training and Research Hospital were retrospectively analyzed to identify individuals who underwent 24-hour ABPM between March 2024 and March 2025 as part of this single-center observational study. Ethical approval for the study was obtained from the Siirt University Ethics Committee (approval no. 2025/01/05/16) and all procedures were performed in compliance with the principles of the Declaration of Helsinki.

During this period, a total of 950 patients aged ≥ 18 years were screened for eligibility. Individuals with incomplete clinical or laboratory data, secondary causes of HT, atrial fibrillation, significant valvular heart disease, end stage chronic kidney disease (CKD) or conditions associated with chronic or acute inflammation were excluded. Patients receiving lipid-lowering, immunosuppressive or systemic anti-inflammatory therapies were not incorporated into the final analytical model. After application of these exclusion criteria, only those with complete datasets and valid ABPM recordings were enrolled.

The final analytical cohort consisted of 241 individuals who fulfilled the eligibility criteria and had complete clinical, laboratory and ABPM data available. Based on ABPM results, participants were classified into two distinct groups: those diagnosed with INH (*n* = 137) and normotensive control subjects (*n* = 104).

### 2.2 Ambulatory blood pressure monitoring

The ABPM over a 24-hour period was conducted with a validated automated oscillometric monitor (Microlife WatchBP O3; Microlife AG, Widnau, Switzerland). BP readings were scheduled every 30 min throughout the daytime and every 60 min during the nighttime. Recordings were deemed acceptable when at least 80% of measurements were successfully obtained.

The following variables were obtained directly from the device-generated report:

1. 24-hour, daytime, and nighttime mean systolic BP (SBP) and diastolic BP (DBP): Calculated as the arithmetic mean of all valid SBP and DBP measurements within the specified time intervals.
2. Mean arterial pressure (MAP): Automatically computed by the device using the formula: MAP = DBP + 1/3 (SBP−DBP).
3. Pulse pressure (PP): Obtained as the difference between SBP and DBP for each reading and averaged over 24-hour, daytime, and nighttime periods.
4. Heart rate (HR): Mean HR values were directly extracted from device recordings.
5. BP load: Defined and calculated by device software as the percentage of readings exceeding conventional daytime (≥ 135/85 mmHg) or nighttime (≥ 120/70 mmHg) thresholds.
6. Nocturnal BP dipping: The device automatically computed dipping percentage using: Dipping (%) = [(Daytime mean BP−Nighttime mean BP)/Daytime mean BP] × 100, and this calculation was applied separately for both systolic and diastolic pressures.

All measurements and derived parameters used in the study were obtained directly from the ABPM report without manual recalculation. The INH was defined in accor-dance with current international guidelines as mean daytime blood pressure < 135/85 mmHg and mean nighttime blood pressure ≥ 120/70 mmHg (1).

### 2.3 Laboratory measurements and Index calculations

Venous blood samples were collected following a minimum fasting period of 8 h. Fasting plasma glucose (FBG), triglyceride, total cholesterol, high-density lipoprotein cholesterol (HDL-C), low-density lipoprotein cholesterol (LDL-C) and C-reactive protein (CRP) concentrations were analyzed using standardized enzymatic assays on an automated Beckman Coulter chemistry platform (Beckman Coulter Inc., Brea, CA, USA). Renal function was assessed by estimating the glomerular filtration rate (eGFR) with the Chronic Kidney Disease Epidemiology Collaboration (CKD-EPI) formula.

The TyG index, which serves as an established proxy for IR, was calculated according to the conventional equation: TyG = ln [fasting triglyceride (mg/dL) × fasting plasma glucose (mg/dL)/2]. To better reflect adiposity-related metabolic risk, a composite TyG-BMI score was generated by multiplying the TyG index by body mass index (BMI). Furthermore, lipid-associated cardiovascular risk was assessed using the atherogenic index of plasma (AIP), calculated as the base-10 logarithm of the triglyceride-to–HDL-C ratio. Non–HDL cholesterol (Non–HDL-C), reflecting the aggregate atherogenic lipid burden, was determined by subtracting HDL-C from total cholesterol. All indices were calculated using fasting laboratory measurements obtained on the same day as ambulatory BP monitoring.

For regression analyses, the TyG index was standardized (ZTyG) to permit interpretation per one standard deviation increment.

### 2.4 Clinical definitions

Diabetes mellitus was diagnosed based on a FBG level of at least 126 mg/dL, a hemoglobin A1c (HgA1c) value ≥ 6.5%, or the use of antidiabetic medication. Participants were classified as having dyslipidemia if they exhibited elevated atherogenic lipid parameters—defined by total cholesterol ≥ 200 mg/dL, LDL-C ≥ 130 mg/dL, triglycerides ≥ 150 mg/dL, or reduced HDL-C levels (< 40 mg/dL for men and < 50 mg/dL for women)—or if they were receiving lipid-lowering therapy. CKD was identified as an eGFR < 60 mL/min/1.73 m^2^. Obesity was diagnosed based on BMI ≥ 30 kg/m^2^. Smoking status and shift work history were obtained from medical records. Obstructive sleep apnea syndrome (OSAS) was defined by prior polysomnographic diagnosis or documented clinical diagnosis. Participants were classified as having coronary artery disease (CAD) if there was a documented previous myocardial infarction, a history of coronary revascularization procedures or the presence of coronary artery stenosis confirmed by angiographic assessment.

### 2.5 Statistical analysis

Continuous data are presented as mean ± standard deviation, whereas categorical data are reported as counts and percentages. Normality of data distribution was evaluated using the Kolmogorov–Smirnov test. Between-group comparisons were conducted with the independent-samples Student's *t*-test for continuous variables, while categorical variables were analyzed using the chi-square or Fisher's exact test, as appropriate.

To determine factors associated with INH, both univariable and multivariable logistic regression models were applied. Covariates demonstrating a *p* value < 0.10 in univariable analyses or those deemed clinically relevant were entered into the multivariable model. Effect estimates are presented as odds ratios (ORs) with corresponding 95% confidence intervals (CIs).

To minimize the risk of multicollinearity, obesity was not included as a separate covariate in the multivariable models, as BMI is already incorporated into the TyG-BMI composite index. Multicollinearity was assessed using the variance inflation factor (VIF) derived from linear regression analysis, and all variables included in the multivariable model demonstrated VIF values below the commonly accepted threshold (range: 1.08–5.88).

The discriminative capacity of TyG-BMI and CRP was assessed using receiver operating characteristic (ROC) curve analysis. Optimal threshold values were identified based on the Youden index, and a *p* value below 0.05 was considered statistically significant.

All analyses were performed using IBM SPSS Statistics version 26.0 (IBM Corp., Armonk, NY, USA).

## 3 Results

### 3.1 Comparison of baseline clinical and metabolic parameters between INH and control groups

A total of 241 patients were included in the analysis, of whom 137 had INH and 104 served as controls. Table 1 provides an overview of the baseline demographic, metabolic, and laboratory features of the study cohort.

**Table 1:** Comparison of demographic, metabolic and laboratory parameters between INH and control groups.

Variables	Control group (*n* = 104) Mean ± SD	INH (*n* = 137) Mean ± SD	*P*-value
Age (years)	56.0 ± 14.1	59.2 ± 13.0	0.067
BMI (kg/m^2^)	26.8 ± 2.9	28.2 ± 2.8	<0.001
TyG index	4.62 ± 0.14	4.80 ± 0.28	<0.001
TyG-BMI	124.0 ± 14.2	135.6 ± 16.4	<0.001
HDL-C (mg/dL)	52.4 ± 6.8	50.7 ± 13.2	0.185
AIP	−0.047 ± 0.13	0.082 ± 0.25	<0.001
LDL-C (mg/dL)	117.7 ± 13.3	113.1 ± 23.9	0.061
Total cholesterol (mg/dL)	193.7 ± 19.6	189.6 ± 35.9	0.260
Non-HDL cholesterol (mg/dL)	141.2 ± 17.6	138.9 ± 32.8	0.477
GFR (mL/min/1.73 m^2^)	91.4 ± 8.7	85.6 ± 18.6	0.002
CRP (mg/L)	1.55 ± 0.99	3.54 ± 1.72	<0.001

Data are presented as mean ± standard deviation. Comparisons between groups were performed using the independent-samples Student's *t*-test. Statistical significance is indicated by *p*-values (*p* < 0.05). INH, isolated nocturnal hypertension; BMI, body mass index; TyG, triglyceride–glucose index; TyG-BMI, triglyceride–glucose–body mass index composite index; AIP, atherogenic index of plasma; HDL-C, high-density lipoprotein cholesterol; LDL-C, low-density lipoprotein cholesterol; CRP, C-reactive protein; GFR, estimated glomerular filtration rate; HT, hypertension.

Individuals with INH exhibited significantly higher BMI values relative to controls (28.2 ± 2.8 vs. 26.8 ± 2.9 kg/m^2^, *p* < 0.001). The INH group exhibited elevated TyG index levels compared with controls (4.80 ± 0.28 vs. 4.62 ± 0.14, *p* < 0.001). Similarly, the composite TyG-BMI index was significantly higher among patients with INH (135.6 ± 16.4 vs. 124.0 ± 14.2, *p* < 0.001). In terms of lipid-related indices, the AIP was significantly elevated in the INH group compared with controls (0.082 ± 0.25 vs. −0.047 ± 0.13, *p* < 0.001). In contrast, serum levels of HDL-C, total cholesterol, non-HDL-C, and LDL-C did not differ significantly between the groups (all *p* > 0.05).

Renal function, as reflected by eGFR, was significantly lower in patients with INH (85.6 ± 18.6 vs. 91.4 ± 8.7 mL/min/1.73 m^2^, *p* = 0.002). Additionally, CRP was substantially higher in the INH group (3.54 ± 1.72 vs. 1.55 ± 0.99 mg/L, *p* < 0.001). Participants in the INH group were generally older than those in the control group; however, this age difference was not statistically significant (59.2 ± 13.0 vs. 56.0 ± 14.1 years, *p* = 0.067).

### 3.2 Comparison of categorical clinical characteristics between INH and control groups

Table 2 summarizes the between-group comparisons of categorical clinical characteristics. The distribution of sex was similar between patients with INH and control group (35.8% vs. 37.5% male, *p* = 0.782). The prevalence of DM was more prevalent among individuals in the INH group than in the control group (24.1% vs. 12.5%, *p* = 0.023). Likewise, the frequency of dyslipidemia was markedly increased among patients with INH (54.7% vs. 28.8%, *p* < 0.001). CKD was also more common in the INH group than in the control group (19.0% vs. 5.8%, *p* = 0.003). Obesity was observed more frequently among patients with INH (24.1% vs. 12.5%, *p* = 0.023). By contrast, the groups did not differ significantly in terms of OSAS, CAD, smoking status, or night-shift work (all *p* > 0.05).

**Table 2:** Comparison of categorical variables between INH and control groups.

Variables	Control group *n* (%)	INH *n* (%)	*χ* ^2^	*P*-value
Sex (male)	39 (37.5%)	49 (35.8%)	0.077	0.782
DM	13 (12.5%)	33 (24.1%)	5.14	0.023
Dyslipidemia	30 (28.8%)	75 (54.7%)	16.13	<0.001
CKD	6 (5.8%)	26 (19.0%)	8.96	0.003
OSAS	8 (7.7%)	18 (13.1%)	1.82	0.177
CAD	16 (15.4%)	30 (21.9%)	1.62	0.203
Smoking	26 (25.0%)	47 (34.3%)	2.43	0.119
Obesity	13 (12.5%)	33 (24.1%)	5.14	0.023
Night-shift work	18 (17.3%)	29 (21.2%)	0.56	0.454

Values are expressed as number (percentage). Chi-square (χ^2^) test or Fisher's exact test was applied where appropriate. Bold values indicate statistically significant results (*p* < 0.05). INH, isolated nocturnal hypertension; HT, hypertension; DM, diabetes mellitus; CKD, chronic kidney disease; OSAS, obstructive sleep apnea syndrome; CAD, coronary artery disease; INH, isolated nocturnal hypertension.

### 3.3 Comparison of ABPM findings between INH and control groups

The ABPM parameters are detailed in Table 3. Patients with INH exhibited significantly higher BP burden across daytime, nighttime, and 24-hour periods. Both daytime and nighttime BP loads were markedly elevated in the INH group as compared to the control group (daytime: 38.3 ± 19.3% vs. 18.3 ± 17.0%; nighttime: 82.7 ± 15.9% vs. 28.9 ± 22.3%; both *p* < 0.001). Similarly, 24-hour mean systolic, diastolic, and MAPs were significantly elevated among individuals with INH (all *p* < 0.001). As expected, nighttime BP values demonstrated the most pronounced differences between groups. Patients with INH had substantially higher nighttime systolic (127.6 ± 7.1 vs. 108.3 ± 7.3 mmHg) and diastolic BPs (72.9 ± 7.5 vs. 62.3 ± 5.2 mmHg) as well as elevated nighttime MAP and PP (all *p* < 0.001). The INH group exhibited higher daytime SBP and DBP values (both *p* < 0.001). In addition, PP was significantly elevated in this group during both daytime and nighttime periods, as well as in the 24-hour period (all *p* < 0.001); whereas no significant intergroup differences were detected with respect to HR parameters, during either daytime, nighttime, or across the 24-hour period (all *p* > 0.05). Nighttime systolic dipping was nearly absent in the INH group (0.17 ± 5.7%) compared with controls (8.6 ± 5.8%, *p* < 0.001) and diastolic dipping was also significantly reduced (3.1 ± 7.3% vs. 12.9 ± 7.0%, *p* < 0.001).

**Table 3:** Comparison of 24-hour ambulatory blood pressure monitoring parameters between groups.

Variables	Control group (*n* = 104) Mean ± SD	INH (*n* = 137) Mean ± SD	*P*-value
Daytime BP load (%)	18.3 ± 17.0	38.3 ± 19.3	<0.001
Nighttime BP load (%)	28.9 ± 22.3	82.7 ± 15.9	<0.001
24-h systolic BP (mmHg)	115.0 ± 7.0	127.8 ± 4.7	<0.001
24-h diastolic BP (mmHg)	68.6 ± 5.4	74.5 ± 6.8	<0.001
24-h MAP (mmHg)	84.0 ± 5.5	92.2 ± 5.2	<0.001
24-h pulse pressure (mmHg)	46.3 ± 5.6	53.4 ± 7.3	<0.001
24-h heart rate (bpm)	73.9 ± 8.5	73.1 ± 9.0	0.515
Daytime systolic BP (mmHg)	118.7 ± 8.1	127.9 ± 5.2	<0.001
Daytime diastolic BP (mmHg)	72.0 ± 5.8	75.3 ± 7.2	<0.001
Daytime MAP (mmHg)	87.6 ± 6.1	92.9 ± 5.7	<0.001
Daytime pulse pressure (mmHg)	46.7 ± 5.9	52.6 ± 7.0	<0.001
Daytime heart rate (bpm)	77.5 ± 9.1	75.8 ± 9.6	0.175
Nighttime systolic BP (mmHg)	108.3 ± 7.3	127.6 ± 7.1	<0.001
Nighttime diastolic BP (mmHg)	62.3 ± 5.2	72.9 ± 7.5	<0.001
Nighttime MAP (mmHg)	77.9 ± 5.8	91.2 ± 6.1	<0.001
Nighttime pulse pressure (mmHg)	45.7 ± 5.9	54.7 ± 8.8	<0.001
Nighttime heart rate (bpm)	67.3 ± 8.6	68.2 ± 8.9	0.413
Nighttime systolic dip (%)	8.6 ± 5.8	0.17 ± 5.7	<0.001
Nighttime diastolic dip (%)	12.9 ± 7.0	3.1 ± 7.3	<0.001

Data are presented as mean ± standard deviation. Comparisons between the isolated nocturnal hypertension group and the control group were performed using the independent-samples Student's *t*-test. Statistical significance is indicated by *p*-values (*p* < 0.05). INH, isolated nocturnal hypertension; BP, blood pressure; SBP, systolic blood pressure; DBP, diastolic blood pressure; MAP, mean arterial pressure; PP, pulse pressure; HR, heart rate; HT, hypertension.

### 3.4 Univariate and multivariate logistic regression analyses for INH

In univariate analyses, DM showed a statistically significant association with higher risk of INH (OR: 2.22, 95% CI: 1.10–4.48, *p* = 0.026). Dyslipidemia was also a strong predictor, with nearly a three-fold increased risk (OR: 2.98, 95% CI: 1.74–5.13, *p* < 0.001). CKD exhibited a strong unadjusted association with INH (OR: 3.83, 95% CI: 1.51–9.68; *p* = 0.005). Elevated CRP levels were related to a four-fold increase in the odds of INH (OR: 4.15, 95% CI: 2.85–6.03; *p* < 0.001), while the AIP demonstrated substantial predictive ability (OR: 20.27, 95% CI: 5.24–78.42; *p* < 0.001). The TyG-BMI index was associated with increased risk on a continuous scale (OR: 1.05 per unit increase, 95% CI: 1.03–1.07; *p* < 0.001), and the ZTyG conferred a 2.55-fold increase in risk per one standard deviation increment (OR: 2.55, 95% CI: 1.80–3.62; *p* < 0.001) (Table 4).

**Table 4:** Univariate logistic regression analysis for predictors of INH.

Variables	OR [Exp(B)]	95% CI	*P*-value
Age	1.018	0.999–1.038	0.068
Male sex	0.928	0.547–1.575	0.782
DM	2.221	1.102–4.476	0.026
Dyslipidemia	2.984	1.736–5.128	<0.001
CKD	3.826	1.512–9.680	0.005
OSAS	1.815	0.757–4.355	0.182
CAD	1.542	0.790–3.011	0.205
Smoking	1.567	0.889–2.762	0.121
Night-shift work	1.283	0.668–2.464	0.454
TyG-BMI	1.052	1.032–1.073	<0.001
AIP	20.265	5.237–78.419	<0.001
CRP	4.147	2.854–6.026	<0.001
ZTyG	2.551	1.798–3.620	<0.001

DM, diabetes mellitus; CKD, chronic kidney disease; OSAS, obstructive sleep apnea syndrome; CAD, coronary artery disease; TyG-BMI, triglyceride–glucose–body mass index composite index; AIP, atherogenic index of plasma; CRP, C-reactive protein; ZTyG, standardized triglyceride–glucose index (per 1 SD increase); INH, isolated nocturnal hypertension.

After controlling for potential confounders in a multivariable logistic regression analysis, the CRP remained the strongest independent predictor of INH (OR: 3.88, 95% CI: 2.61–5.76; *p* < 0.001). Moreover, TyG-BMI remained an independent predictor, with each unit increase being associated with a 3% higher odds of INH (OR: 1.03, 95% CI: 1.00–1.06; *p* = 0.034) (Table 5). The fully adjusted model exhibited satisfactory calibration and discriminative performance, supported by a nonsignificant Hosmer–Lemeshow test result (*p* = 0.733), a Nagelkerke R^2^ value of 0.598, and an overall correct classification rate of 83.0%.

**Table 5:** Multivariate logistic regression analysis for predictors of INH.

Variable	B	SE	Wald	*P-*value	OR [Exp(B)]	95% CI for OR
Age	0.015	0.013	1.258	0.262	1.015	0.989–1.042
DM	–0.285	0.606	0.222	0.638	0.752	0.229–2.466
Dyslipidemia	0.674	0.457	2.177	0.140	1.963	0.801–4.808
CKD	0.447	0.706	0.400	0.527	1.563	0.392–6.243
TyG-BMI	0.030	0.014	4.499	0.034	1.030	1.002–1.059
AIP	–2.041	1.999	1.042	0.307	0.130	0.003–6.534
CRP	1.355	0.202		<0.001	3.878	2.609–5.763
ZTyG	0.919	0.525	3.056	0.080	2.506	0.895–7.019
Constant	–7.726	2.072	13.906	0.000	—	—

DM, diabetes mellitus; CKD, chronic kidney disease; TyG-BMI, triglyceride–glucose–body mass index; AIP, atherogenic index of plasma; CRP, C-reactive protein; ZTyG, standardized triglyceride–glucose index; INH, isolated nocturnal hypertension.

### 3.5 Discriminative performance of TyG-BMI and CRP

**Figure 1:**
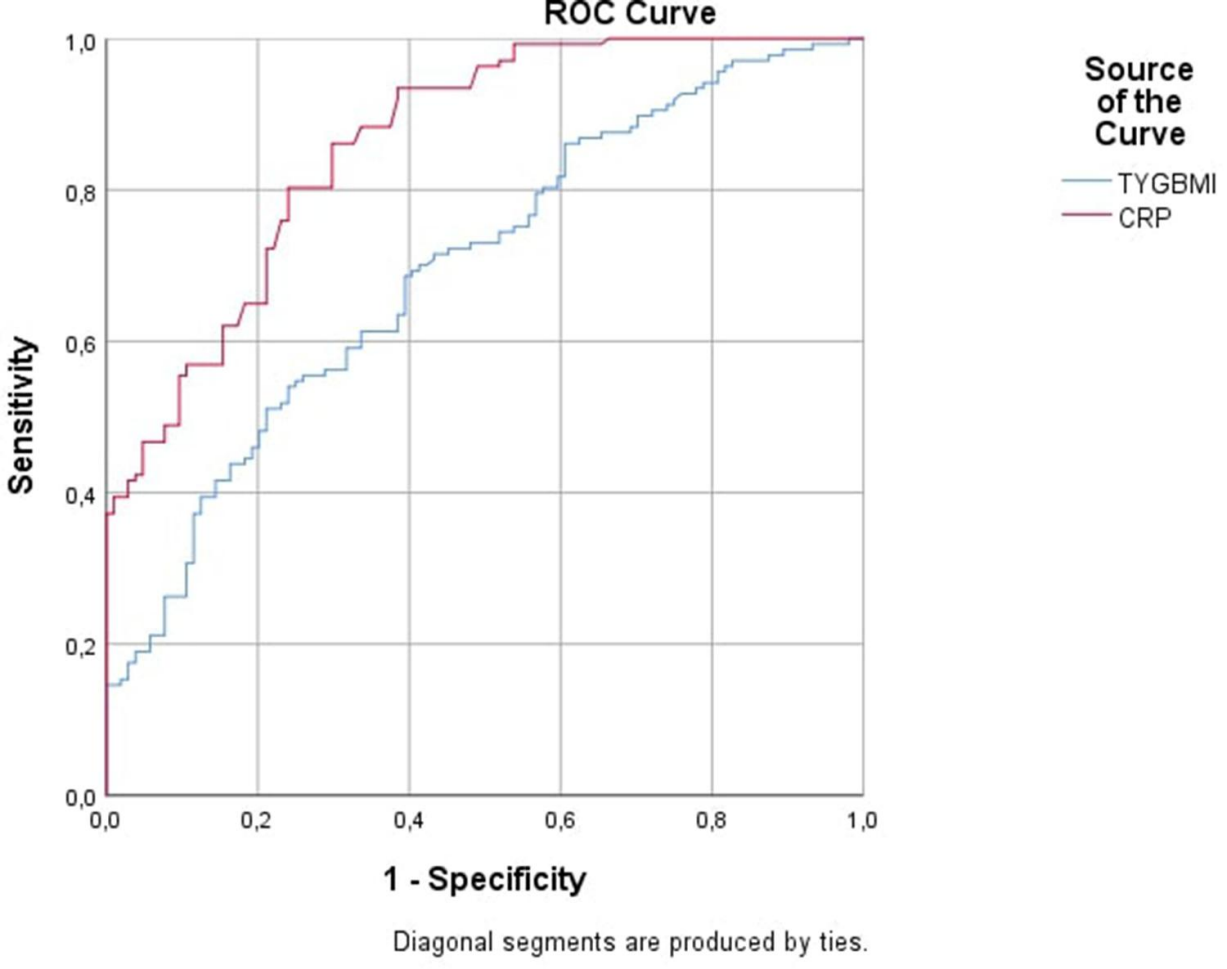
ROC curves of TyG-BMI and CRP for predicting INH.

Receiver operating characteristic curve analysis was used to assess the predictive performance of TyG-BMI and CRP in identifying INH (Table 6). The TyG-BMI demonstrated a modest but significant diagnostic performance, with an AUC of 0.696 (95% CI: 0.630–0.762; *p* < 0.001). In contrast, CRP exhibited a strong discriminative capacity, with an AUC of 0.861 (95% CI: 0.816–0.906; *p* < 0.001) (Figure 1). Confidence intervals for sensitivity and specificity corresponding to the optimal cut-off values for both TyG-BMI and CRP are presented in Table 6.

**Table 6:** Diagnostic performance of TyG-BMI and CRP for predicting INH.

Variable	AUC (95% CI)	*P*-value	Cut-off	Sensitivity (%) (95% CI)	Specificity (%) (95% CI)	PPV (%)	NPV (%)	Youden index
TyG-BMI	0.696 (0.630–0.762)	<0.001	129.2	63.5 (55.1–71.1)	61.5 (52.0–70.3)	68.5	56.2	0.250
CRP (mg/L)	0.861 (0.816–0.906)	<0.001	2.285	80.3 (72.8–86.2)	76 (67.0–83.4)	81.4	74.6	0.563

AUC, area under the curve; CI, confidence interval; PPV, positive predictive value; NPV, negative predictive value; TyG-BMI, triglyceride–glucose–body mas index; CRP, C-reactive protein; INH, isolated nocturnal hypertension.

## 4 Discussion

The present analysis examined the relationship between INH and a range of metabolic as well as inflammatory parameters. Individuals with INH demonstrated significantly higher TyG-BMI values and CRP levels compared with controls, and both markers remained independently associated with INH. The strong discriminatory performance of CRP further highlights the role of chronic inflammation in nocturnal BP elevation. Taken together, the evidence points to INH as a phenotype shaped by metabolic and inflammatory processes, rather than merely a hemodynamic variation.

The cut-off values identified for TyG-BMI and CRP were statistically derived using the Youden index to optimize sensitivity and specificity within the study population. These thresholds should be interpreted as hypothesis-generating rather than definitive clinical cut-offs and require validation in larger, prospectively designed cohorts before being applied in routine clinical practice. To our knowledge, there are no widely accepted TyG-BMI or CRP cut-off values established for INH in prior studies, underscoring the exploratory nature of the present findings.

Metabolic dysregulation has long been implicated in abnormal circadian BP behavior. IR contributes to endothelial dysfunction, oxidative stress, sympathetic activation, and vascular stiffness (14–16), all mechanisms that may disproportionately influence nighttime BP levels. Our observation that TyG-BMI was more strongly associated with INH than TyG alone aligns with evidence showing that TyG-BMI better reflects composite metabolic burden and predicts metabolic syndrome and cardiovascular events more accurately than TyG (17, 18). In large population-based studies, higher TyG-BMI levels have been remains independently correlated with higher systolic and diastolic BP values and a higher risk of HT, even after multivariable adjustment (19). Because renin–angiotensin–aldosterone system (RAAS) activity increases during nocturnal sleep, bedtime metabolic and hemodynamic disturbances may exert a disproportionately greater influence on nighttime BP regulation. Evidence from a randomized trial by Hermida et al. demonstrated that nocturnal RAAS blockade improves glucose and insulin tolerance and reduces the risk of new-onset DM (20). This mechanism aligns with our findings that TyG-BMI and CRP were strongly associated with INH, suggesting that individuals with higher metabolic or inflammatory burden may experience amplified nocturnal RAAS activation, thereby promoting the development of INH.

Although the AIP demonstrated a strong association with INH in univariable analysis, this association was attenuated after adjustment for interrelated metabolic and inflammatory markers in the multivariable model. This finding likely reflects shared metabolic pathways rather than model overfitting, as AIP, TyG-BMI, and related lipid indices capture overlapping aspects of cardiometabolic risk. In this context, composite indices such as TyG-BMI may more comprehensively reflect the integrated metabolic burden relevant to nocturnal BP dysregulation.

In addition to these mechanisms, adiposity itself—through heightened sympathetic nervous system activation and increased renal sodium reabsorption—plays a key role in nocturnal BP regulation. Obesity-related HT has been strongly linked to increased renal sympathetic nerve activity, which promotes sodium and volume overload (21). In parallel, visceral adiposity has been implicated in triggering activation of the sympathetic nervous system as well as the RAAS, structural and functional renal changes, and impaired natriuresis (22–24). Furthermore, IR coexisting with obesity may exacerbate renal sodium reabsorption and vascular dysfunction (25). In this context, TyG-BMI's superior predictive value for INH likely stems from its capacity to capture both metabolic dysregulation and adiposity-mediated neurohumoral and renal mechanisms.

Inflammation represents another critical pathway contributing to nocturnal BP elevation. Recent data indicate that low-grade systemic inflammation disrupts endothelial nitric oxide signaling, increases arterial stiffness, and enhances neurohumoral activation, all of which may favor nocturnal rather than daytime BP increases (26–28). In line with this framework, accumulating clinical evidence indicates that increased concentrations of high-sensitivity CRP are linked to abnormal nocturnal BP patterns, including attenuated nocturnal dipping and higher nighttime systolic BP, supporting a role for inflammation in circadian BP dysregulation (29). CRP is widely regarded as a reliable indicator of low-grade systemic inflammatory activity and has repeatedly been associated with impaired endothelial function, increased sympathetic drive, and heightened renal sodium reabsorption (30–32). These mechanisms plausibly explain why CRP demonstrated strong predictive performance in our study, suggesting that inflammatory activation may exert a disproportionate effect during nighttime, when autonomic balance and vascular tone are more vulnerable to dysregulation. Nevertheless, given the retrospective nature of the study, residual confounding and selection bias cannot be entirely excluded, and the observed discriminatory performance of CRP should therefore be interpreted with caution. Prospective studies are warranted to confirm the robustness and clinical applicability of CRP as a marker of INH.

Metabolic impairment and inflammation frequently coexist, forming a reciprocal feedback loop that promotes circadian BP abnormalities. IR enhances inflammatory cytokine production, while inflammation further aggravates metabolic dysfunction by altering adipokine signaling, impairing mitochondrial function, and promoting oxidative stress (27, 33–35). This bidirectional interaction has been proposed as a core mechanism linking metabolic stress to disrupted nocturnal BP patterns. Our findings support this integrated model, as both TyG-BMI and CRP—markers reflecting metabolic and inflammatory burden—were strongly associated with INH, reinforcing the view that INH represents a cardiometabolic–inflammatory phenotype rather than an isolated hemodynamic alteration.

### 4.1 Limitations

This study is subject to certain limitations. In particular, its retrospective observational design precludes causal interpretation and leaves open the possibility that unmeasured or residual confounders may have affected the observed associations. Specifically, behavioral and lifestyle-related factors such as sleep quality, dietary sodium intake, physical activity levels, and autonomic nervous system activity could not be systematically assessed within the scope of the available dataset and may have influenced nocturnal BP patterns. We did not assess additional inflammatory cytokines, autonomic function parameters, or endothelial biomarkers, which might have provided further mechanistic insight into nocturnal BP regulation. Future prospectively designed studies incorporating broader inflammatory biomarker panels, including cytokines such as interleukin-6 and tumor necrosis factor-α, may further elucidate the mechanistic links between inflammation and nocturnal BP dysregulation. Obesity was not included as a separate variable in the multivariate model to avoid multicollinearity, given that adiposity is already incorporated into the TyG-BMI composite index. In addition, although 24-hour ABPM is the gold standard for diagnosing INH, more advanced techniques such as beat-to-beat BP monitoring could have provided a more detailed assessment of short-term nocturnal BP variability and autonomic regulation. Finally, conducting the study at a single institution may limit the external validity of the findings. Differences in ethnic background, regional lifestyle patterns, and healthcare system–related factors may influence the applicability and performance of TyG-BMI and CRP across broader populations. Accordingly, larger-scale, prospectively designed investigations incorporating standardized assessments of lifestyle factors and autonomic function, and involving multiple centers, are required to both substantiate these results and further elucidate the underlying biological mechanisms.

### 4.2 Future directions

From a clinical standpoint, the results of the present analysis carry noteworthy clinical and scientific implications. First, INH is frequently missed in routine practice because patients often present with normal daytime BP. Incorporating simple biomarkers such as TyG-BMI and CRP into initial cardiovascular risk assessment may help clinicians identify individuals who merit ambulatory BP monitoring. Second, because CRP demonstrated particularly strong predictive performance, inflammation-focused risk stratification may offer additional value beyond traditional metabolic parameters. In conclusion, conceptualizing INH as a cardiometabolic and inflammatory entity may support the timely detection of individuals at elevated risk and encourage the initiation of preventive strategies, including lifestyle modification and targeted pharmacological approaches aimed at metabolic and inflammatory processes.

Nevertheless, these findings should be regarded as hypothesis-generating, and TyG-BMI and CRP should not yet be considered for routine clinical screening or guideline-based risk stratification of INH without confirmation from prospective studies.

## 5 Conclusion

This study demonstrates that TyG-BMI and CRP are significantly associated with INH, with CRP showing particularly strong independent predictive value. These results emphasize the significant role that metabolic imbalances and minor inflammation play in the emergence of this nighttime BP pattern. Incorporating such simple and widely available biomarkers into early clinical assessment may help identify individuals who require ambulatory BP monitoring. Additional research is required to confirm the reliability of these findings and to clarify whether biomarker-based screening for INH could enhance detection in routine clinical practice.

## Data Availability

The raw data supporting the conclusions of this article will be made available by the authors, without undue reservation.
